# Molecular versatility during pluripotency progression

**DOI:** 10.1038/s41467-022-35775-4

**Published:** 2023-01-05

**Authors:** Giacomo Furlan, Aurélia Huyghe, Noémie Combémorel, Fabrice Lavial

**Affiliations:** 1 Cellular reprogramming, stem cells and oncogenesis laboratory - Equipe labellisée La Ligue Contre le Cancer – LabEx Dev2Can - Univ Lyon, Université Claude Bernard Lyon 1, INSERM 1052, CNRS 5286, Centre Léon Bérard, Cancer Research Center of Lyon, Lyon, 69008 France; 2 Present Address: Lunenfeld-Tanenbaum Research Institute, University of Toronto, Toronto, ON Canada

**Keywords:** Embryonic stem cells, Self-renewal, Stem-cell differentiation

## Abstract

A challenge during development is to ensure lineage segregation while preserving plasticity. Using pluripotency progression as a paradigm, we review how developmental transitions are coordinated by redeployments, rather than global resettings, of cellular components. We highlight how changes in response to extrinsic cues (FGF, WNT, Activin/Nodal, Netrin-1), context- and stoichiometry-dependent action of transcription factors (Oct4, Nanog) and reconfigurations of epigenetic regulators (enhancers, promoters, TrxG, PRC) may confer robustness to naïve to primed pluripotency transition. We propose the notion of Molecular Versatility to regroup mechanisms by which molecules are repurposed to exert different, sometimes opposite, functions in close stem cell configurations.

## Abstract (web summary)

During development the embryo must balance lineage specification against the preservation of plasticity using a limited molecular toolkit. In this Perspective, the authors propose Molecular Versatility as a paradigm for grouping molecular mechanisms that are repurposed through development to exert distinct functions.

## Introduction

In contrast to the all-or-none view of the unidirectional differentiation of a stem cell into terminally specialised derivatives, the development of multicellular organisms involves developmental transitions that often generate plastic and reversible cellular intermediate states. To achieve these transitions, embryonic cells need to integrate complex sets of external cues and respond by precise epigenetic/transcriptomic changes that ensure the stepwise and coordinated lineage segregation^1^. Mouse peri-implantation development constitutes a prototypical example of such dynamic transitions during which the naïve pluripotent cells of the early epiblast transit to formative and primed pluripotent identities prior to differentiating. During this process, the reconfigurations of the epigenetic/transcriptional programs permit the emergence of distinct molecular features, including the transient competence to form primordial germ cells (PGCs)^2^. However, the embryonic cells also maintain their ability to self-renew and sustain the expression of certain core pluripotency transcription factors (TFs)^2,3^. Therefore, a critical challenge for peri-implantation cells is to robustly acquire novel features while preserving their stemness in constrained developmental space and time. In this Perspective, we exploited recent findings in the pluripotency field to question whether these cellular transitions involve exclusively complete molecular resettings (–e.g. extinction of a full transcriptional/epigenetic program and induction of a non-overlapping new one), as traditionally proposed for differentiation processes^4^, or also alternative mechanisms. Hence, we herein compiled convergent findings depicting how a large battery of molecules are efficiently repurposed to exert strikingly different, sometimes opposite, functions in these closely-related stem cell configurations. We thus propose more broadly that pluripotency progression is also ensured by the fine-tuned redeployment, complementary to the resetting, of a large repertoire of intrinsic components of the cell, ranging from the plasma membrane to the chromatin. Far from a semantic argument, we propose the notion of Molecular Versatility (see Box 1) to regroup these mechanisms that, we believe, might have been evolutionary co-opted to confer robustness to developmental transitions.

**Fig. 1:**
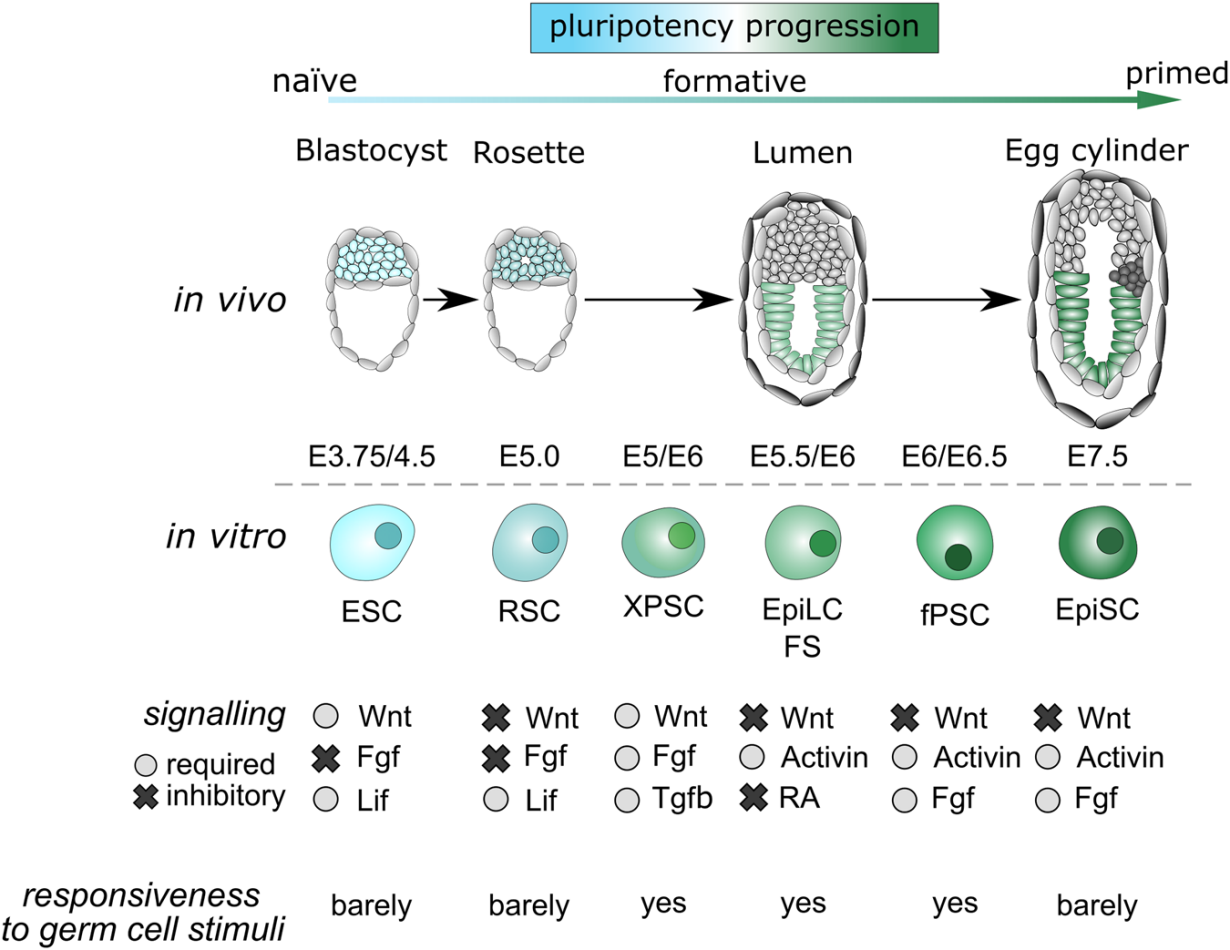
A continuum of transitional states defines pluripotency progression. The schematic diagram depicts the embryonic stages and the corresponding embryo-derived cell types. It also summarises the signalling pathways that sustain the embryo-derived stem cells in vitro and their ability to respond to germ cell inductive signals. ESC: Embryonic Stem Cell, RSC: Rosette Stem Cells^13^, XPSC: X Pluripotent Stem Cells^15^, EpiLC: Epiblast-like Cells^12^, FS: Formative Stem cells^137^, fPSC: Formative Pluripotent Stem Cells^16^, EpiSC: Epiblast Stem Cells.

During peri-implantation development, pluripotency does not correspond to a unique and fixed cellular state but rather encompasses multiple evolving cellular configurations (Fig. 1)^5^. These closely-related cellular states share the ability to self-renew and the expression of a set of core TFs, as reviewed elsewhere^3,6^. Yet, they undergo profound changes in their ability to respond to extrinsic cues, that allow the establishment of the pluripotent compartment of the early and late epiblasts^7^. Before implantation (embryonic day E4.5), mouse epiblast cells reside in a naïve state, which progressively transitions to formative (E5.5–E6.5) and primed (E6.5–E7.5) configurations upon implantation (Fig. 1)^8^. These transitions, which are crucial for germ layer formation, prepare embryonic cells for lineage specification while maintaining stemness. The regulatory mechanisms controlling these transitions are complex to decipher in vivo due to the limited access to and quantity of biological material at these embryonic stages. Yet they can be dissected using tractable and faithful in vitro models of embryo-derived stem cells. These models were initially classified dichotomously with mouse embryonic stem cells (ESCs) reflecting the naïve epiblast^9^, and epiblast stem cells (EpiSCs) resembling the primed epiblast of the anterior primitive streak^10,11^. Intermediate cellular states were captured, such as the transient Epiblast-like cells (EpiLCs)^12^. More recently, Rosette-like stem cells (RSCs)^13^ and pluripotent stem cells harbouring features of formative pluripotency were captured using various culture conditions^14–16^ (Fig. 1). Like their in vivo counterparts, these closely related cell types are characterised by distinct developmental properties, signalling requirements, transcriptomes and epigenomes. Notably, naïve ESCs are unresponsive to germ cell inductive stimuli, in contrast to EpiLCs and formative stem (FS) cells that acquired such competence. Yet, EpiSCs already lost responsiveness to PGC induction (Fig. 1). Of note, the transition from naïve to formative pluripotency can also be explored in vitro using fluorescent reporters of the naïve marker Rex1/Zfp42 in ESCs^17^.

Therefore, during pluripotency progression, an apparently contradictory challenge for embryonic cells is to retain stemness while learning to respond accurately to lineage specifying cues^3^. A critical underlying question is how such challenge is addressed rapidly (in few days) and precisely at the molecular level. Recent attempts combining CRISPR/Cas9 gene disruption, large-scale transcriptomics and computational systems biology revealed that the transition from naïve to formative pluripotency is not based on the action of few master TFs but rather the product of multiple crosstalking components linking at least four signalling inputs with epigenetic and TF networks to rewire embryonic cells^18,19^. Here, we selected in the recent literature a battery of molecular mechanisms by which signalling pathways, TFs and epigenetic regulators are repurposed to reach this objective. In addition to the established notion that molecules can exert strikingly different functions in unrelated cell types, we propose the notion of Molecular Versatility to regroup these molecular mechanisms that specifically take place in similar or closely related stem cell configurations during peri-implantation development (Box 1).

Box 1 Glossary **Molecular Versatility:** This notion regroups the mechanisms by which molecules (including members of signalling pathways, TFs and epigenetic regulators) exert strikingly different, sometimes opposite, functions in similar or closely-related stem cell configurations (continuum of cellular intermediate states, early progenitors). This notion differs from the concept that molecules exert different functions in completely unrelated cell types. Molecular Versatility rather relates to mechanisms occurring during cellular transitions in constrained time and space.

**Cellular identity:** Cellular identity can be defined in multiple ways and recent advances in single-cell analyses have complicated this definition by unveiling substantial transcriptional heterogeneity among cells within traditionally defined cell types^138^. In this Perspective, cellular identity describes the transcriptional and epigenetic programs that define a given cell type in homeostatic (non-injured, non-inflammatory) conditions.

## Redeploy signalling pathways

The maintenance of the naïve, formative and primed pluripotent states in vitro heavily relies on signalling pathways^20^. It has to be noted that some of these signals may be dispensable for embryo development, except under certain conditions (diapause), as reported for LIF^21^. Naïve ESCs self-renew in a metastable state in the presence of LIF and BMP4^22^ whereas the activation of the WNT pathway prevents their transition towards primed EpiSC^23^. The combined use of chemical inhibitors (2i) to suppress FGF/MAPK (via MEK1/2) and activate WNT (via GSK3 α/β) signalling instructs a ground state of pluripotency^24^ that can be partially mimicked by the Netrin-1 signalling pathway^25^. In contrast, the combined inhibition of FGF/MAPK and WNT signalling results in the capture and maintenance of RSCs in a self-renewing intermediate naïve-primed state^13^. Moreover, EpiLCs, FS and EpiSCs rely on Activin/Nodal and FGF/MAPK to maintain their undifferentiated state^10,12^ (Fig. 1). In line with the Molecular Versatility notion, we selected below key mechanisms that allow closely related embryonic cells to integrate differently these signalling molecules.

### The FGF/MAPK signalling pathway: a tight control of ERK1/2 activity

**Fig. 2:**
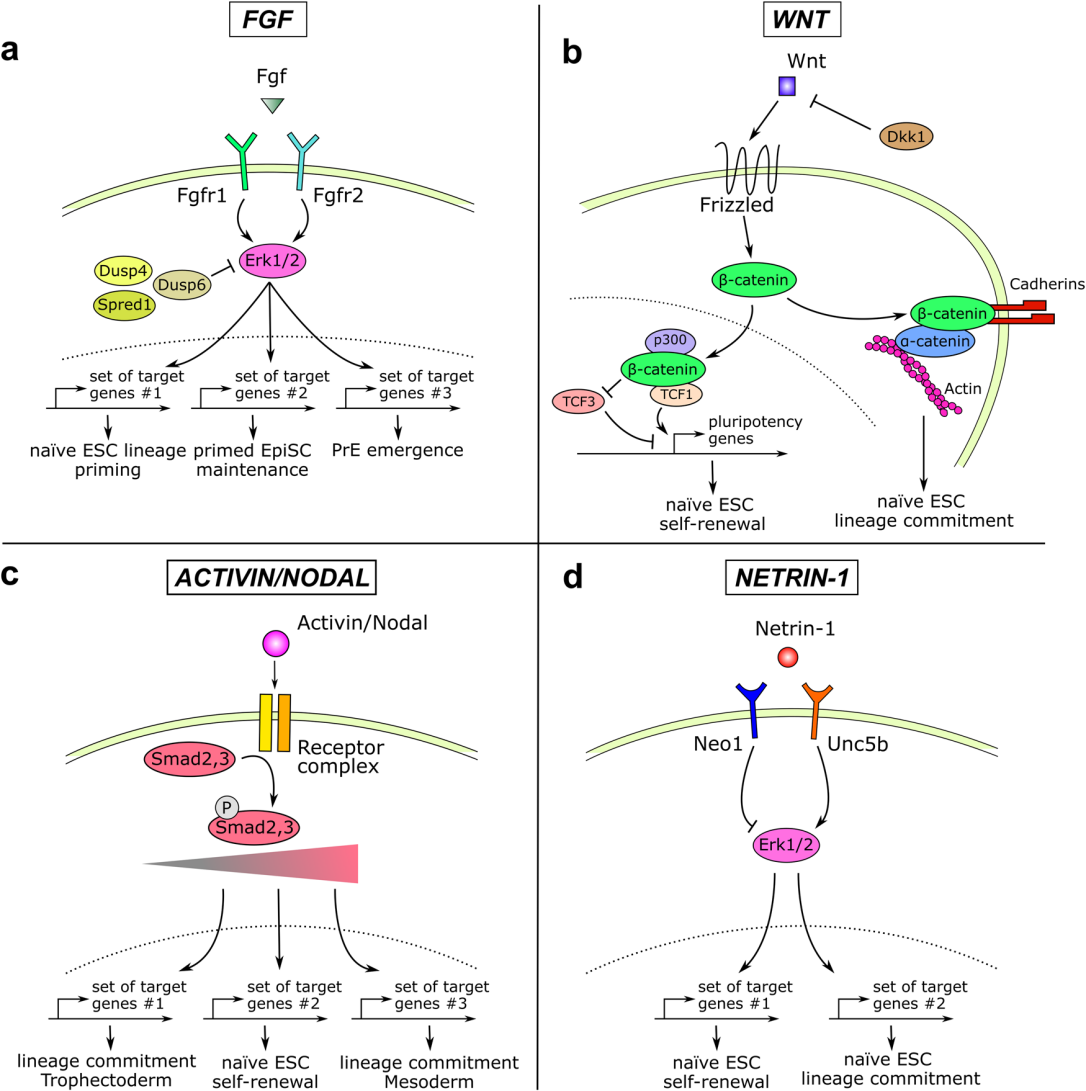
Molecular Versatility of signalling pathways. **a** FGF/MAPK signalling. The integration of the FGF signal is modulated by the differential expression of the FGFR1 and FGFR2 receptors. Such integration will lead to various degrees of ERK1/2 activation that will subsequently activate different sets of target genes. **b** Wnt signalling. The activation of the pathway will promote stem cell self-renewal or lineage commitment. These versatile effects are related to the bifunctional activity of β-catenin in the nucleus and in the cytoplasm. It has to be noted that TCF1 and TCF3 also behave antagonistically on pluripotency genes and ESC self-renewal. **c** Activin/Nodal signalling. Activin/Nodal signalling leads to a various degree of Smad2 activation. This precise degree triggers the regulation of different sets of genes and therefore of different cell fate decisions in ESCs. **d** Netrin-1 signalling. The Netrin-1 molecule has opposite effects on ESC fate by promoting self-renewal or lineage commitment. This Molecular Versatility is controlled by the stoichiometry of its receptors Neo1 and Unc5b that leads to ERK1/2 activity induction or repression.

A first prototypical illustration of Molecular Versatility is associated with the FGF/MAPK pathway, and in particular with its receptors FGFR1/FGFR2 and its effectors ERK1/2. The activation of the pathway is initially instrumental for the emergence of the extraembryonic trophectoderm (TE) and primitive endoderm (PrE) lineages^26^. Of interest for the Perspective, the pathway is required for naïve ESCs to acquire competence to form somatic cell types^12,27,28^ but, in striking contrast, the pathway then sustains pluripotency in EpiLCs/FS/EpiSCs^10,29^. Therefore, the response to FGF must be rapidly and efficiently modified in these closely-related stem cell configurations to ensure those strikingly different effects (Fig. 2a). Even if the precise molecular mechanisms are not known, we describe below some related findings that might contribute to the phenomenon.

A first element of response lies in the combined use of the FGF receptors on epiblast (Epi) and PrE progenitors. FGFR2 was long considered to be the main receptor actively promoting PrE emergence as the onset of its expression was detected by E3.5 in PrE-biased cells^30,31^. However, this sole FGF4/FGFR2 axis was not sufficient to explain the different functions exerted by the pathway in PrE emergence and Epi maturation. Quantitative single-cell-resolution imaging, combined with gene expression profiling of FGFR mutant/knock-in embryos, elegantly revealed how the stoichiometry of the receptors FGFR1 and FGFR2 is at least partially responsible for these differential actions^28,32^. While Epi-biased cells express solely FGFR1, PrE-biased blastomeres co-express FGFR1 and FGFR2. This variation ensures that apparently similar cells respond differently to the same ligand FGF4. The autocrine FGF4/FGFR1 axis is required for epiblast cells to mature towards the primed state while the paracrine FGF4/FGFR1/FGFR2 signalling stabilises the fate of PrE cells. In line with this, the activation of FGF/MAPK signalling in PrE- and Epi-biased cells was described to trigger the activation of different sets of target genes^28^. Accordingly, the recent development of ERK-kinase translocation reporter, enabling live quantification of ERK1/2 activity at the single-cell resolution in vivo, revealed that, prior to lineage specification, PrE progenitors already harbour higher ERK1/2 activity than Epi progenitors^33^. Of note, the sensitivity of embryonic cells to FGFs can also be mediated by the differential expression of FGF/MAPK inhibitors such as Dusp4, Dusp6, and Spred1, and the induction of negative feedback loops^34^ (Fig. 2a). The modulation of FGF availability and engagement on its receptors via heparan sulphate proteoglycans^35^ constitute other regulatory mechanisms, as well as the distinct usage of intracellular pathways (Jak/Stat, PLCγ, PI3K and MAPK), as reviewed elsewhere^36,37^.

Another interesting example of Molecular Versatility comes from works on the MEK/ERK factors that were found to exert dual functions in naïve ESCs, respectively promoting (i) self-renewal and (ii) lineage commitment. On the one hand, the chemical inhibition of MEK/ERK is known to constrain ESCs differentiation and to be instrumental for sustaining the ground state of naïve pluripotency^24^. However, on the other hand, Chen and colleagues demonstrated, using an inducible KO of both ERK1 and ERK2, that such complete depletion led to rapid genomic instability and compromised ESC self-renewal^38^. In line with this, the prolonged MEK inhibition was also shown to alter ground state ESC genomic stability^39^. Therefore, a precise dosage of ERK1/2 activity (intensity, duration) seems to dictate very different outcomes in naïve cells. An alternative way to tightly control the effect of ERK1/2 activity in ESCs came from an elegant study from the Brickman lab. The authors reported that the pro-lineage commitment activity of ERK1/2 is reversible, to a certain extent. Following stimulation, ERK1/2 was found to regulate first the transcription and enhancer activity of genes without inducing any changes in the binding of pluripotency TFs but rather by modifying the association of RNA polymerase II and cofactors such as Mediator components with the chromatin. Later on, if the stimulation persists, ERK1/2 finally dismantles pluripotency by directly phosphorylating/destabilising TFs such as Klf4/Nanog and RNA polymerase II at Ser5 residue. In contrast, if stimulation stops, the reduced ERK1/2 activity restores the normal expression of pluripotency proteins. This mode of regulation indicates how the activation of ERK1/2 activity induces a transient window of lineage commitment during which reversion to naïve pluripotency is preserved^40^. Altogether, these examples underline various ways by which embryonic cells may modulate their response to FGF signals.

### The WNT signalling pathway: bi-functional properties of β-catenin

The WNT signalling pathway also exerts strikingly different functions in naïve and primed pluripotent stem cells. While its activation contributes to maintaining naïve pluripotency in ESCs^41^, it subsequently triggers exit from primed pluripotency and mesendoderm differentiation in EpiSCs^42^. We highlight below some molecular mechanisms that may allow embryonic cells to precisely modulate their responsiveness to Wnt during pluripotency progression.

First, the bi-functional nature of the effector β-catenin per se allows it to exert dual functions in promoting (i) self-renewal but also (ii) priming for differentiation (Fig. 2b). β-catenin harbours transactivating functions in the nucleus but it is also a component of adherens junctions in the cytoplasm. In the nucleus, β-catenin contributes to sustaining naïve pluripotency by triggering TCF3 inactivation by a plethora of mechanisms, ranging from the control of TCF3 protein binding/stability to the epigenetic regulation of its promoter or transcript by miRNA^43,44^. More recently, nuclear β-catenin was also found to strengthen the robustness of the transcriptional apparatus by supplying transcriptional co-regulators (such as BRD4, CDK9, Mediator, Cohesin and p300) at pluripotency loci^45^. In contrast in the cytoplasm, β-catenin contributes to controlling cell adhesion by connecting cadherins through α-catenin to the actin cytoskeleton^46,47^. The use of a TCF-signalling-defective-β-catenin variant demonstrated that its cell-adhesion functions are critical for endodermal and neuronal differentiation^46^.

Additional layers of regulation contribute to the Molecular Versatility of the Wnt pathway, such as the repertoire of the TCF TFs. In ESCs, TCF1 and TCF3 were shown to exert opposite functions, respectively promoting and destabilising the pluripotent network by competing for DNA binding through virtually identical HMG domains^43,48,49^. In addition, the noncanonical Wnt ligand Wnt5a was shown to exert opposite actions, namely activating or repressing β-catenin/TCF signalling, depending on the receptor it bound to (Ror2 or Frizzled4), indicating that the repertoire of receptors controls the response to WNT pathway^50^. This phenomenon will be discussed in more detail in the Netrin-1 signalling pathway section. Finally, the secretion of WNT antagonists, such as Dkk1 by the PrE^13^, and the existence of auto-activating loops, constitute alternative strategies to modulate response to WNT signalling^51^.

### The Activin/Nodal signalling pathway: a dose-dependent switch of Smad binding

Activin and Nodal are morphogens of the TGFβ superfamily that direct differential stem cell fate decisions in a dose- and distance-dependent manner^52^. During early embryonic development, the pathway is responsible for the specification of mesoderm, endoderm, node, and mesendoderm. In contradiction to this function in differentiation, the pathway also plays important roles in ESCs and EpiSCs (Fig. 2c). Indeed, recombinant Activin A is used in vitro for the continued propagation and expansion of EpiSC^10,29^ and Activin/Nodal were found to promote ESC proliferation^53,54^. In that context, Lee and Colleagues proposed a fascinating model of how ESCs interpret and respond differentially to Activin/Nodal dosage^55^. They reported that, whereas the endogenous level of Activin/Nodal signaling is required for ESC self-renewal, its perturbation leads to an exit from pluripotency towards different paths: mesendoderm induction at high signalling level and TE differentiation at low signalling level. Mechanistically, phospho-Smad2, the primary downstream transcriptional effector of the Activin/Nodal pathway, was found to bind and regulate in a dose-dependent manner distinct subsets of target genes, including the Oct4 promoter. This dose-dependent switch of binding by phospho-Smad2 provides a molecular basis for the versatile functions of Activin/Nodal in ESCs^55^.

### The Netrin-1 signalling pathway: modulating ligand and receptors stoichiometry

The Netrin-1 signalling pathway also permits to highlight Molecular Versatility during embryonic development. Netrins are a conserved family of laminin-related molecules that act chiefly through receptors of two distinct families: the deleted in colorectal cancer and UNC-5 families^56^. We recently revealed that the ligand Netrin-1, initially described as an axon guidance cue, exerts opposite effects on naïve ESC self-renewal, by activating or repressing ERK1/2, when the relative stoichiometry of its receptors Neogenin1 (Neo1) and Unc5b is experimentally modulated (Fig. 2d)^25^. Therefore, the repertoire of receptors allows the same ligand to trigger different responses in ESCs. Interestingly, the same receptor can also exert alternative functions depending on the ligand it binds to. Indeed, in the same settings, the Neo1 receptor was found to induce chemoattraction via Netrin-1 but chemorepulsion via the ligand Repulsive guidance molecule a (Rgma)^57^.

More generally, these results questioned why, rather than using single ligand and receptor couples to dictate different outcomes, signalling pathways comprise multiple ligand and receptor variants that interact promiscuously to generate large sets of distinct signalling complexes. The use of redundant ligands and receptors was proposed to confer regulatory flexibility^58^ or robustness to biological processes^59^ but this apparent redundancy recently appeared as a strong way to generate diverse and specific cellular responses. As an example, in an attempt to understand how the ligands Netrin-1 and Rgma cross-talk to transduce their signals, Robinson and colleagues reported that their simultaneous binding to Neo1 does not lead to competition for binding but to the formation of a ternary super-complex that globally diminishes their functional outputs^60^. Moreover, by combining theoretical and experimental approaches, the Elowitz laboratory showed how cells perceive absolute but also relative concentrations of multiple ligands to compute multi-dimensional response profiles. In this model, by modifying the stoichiometry of receptors, cells modulate the computations to perform, and therefore the responses to provide to the same combination of ligands^61^. Mathematical modelling even revealed that such multi ligand/receptor system outperforms seemingly more specific one-to-one signalling architectures in terms of diversity and specificity of responses^62^. In line with this, this section highlighted different mechanisms by which embryonic cells modulate their response to their surrounding signals.

## Redirect transcription factors

Naïve, formative and primed stem cells are characterised by the shared expression of TFs such as Oct4 and Sox2^10,12,13^, raising fascinating questions about how distinctive properties can be established in the presence of an overlapping set of master TFs. In line with the Molecular Versatility notion, a large fraction of pluripotency TFs were initially found to exert opposite functions in ESCs by promoting (i) self-renewal at endogenous levels but (ii) germ layer formation when ectopically expressed, including Sox2 (neurectoderm)^63^ and Esrrb/Tbx3 (endoderm)^64,65^. A limitation of these studies is that overexpression may show non-physiological activity of TFs as a result of non-specific binding and/or sequestration of factors. However, in the case of Oct4, its endogenous level was also reported to destabilise self-renewal, as illustrated by the fact that Oct4± heterozygous ESCs harbour a more robust pluripotent state than WT ESCs^66^. Therefore, based on these findings, Loh and Lim proposed to consider naïve pluripotency TFs as “pure lineage specifiers” and pluripotency as an inherently precarious condition in which these TFs compete to generate mutually exclusive lineages. This intriguing concept was slightly contrasting with the model proposed by A. Smith of a “ground” state of pluripotency in which pluripotency TFs rather cooperate to constrain the fluctuations provided by opposing differentiation programs^24,67–69^. Of note, this notion of TF battles was initially described in the hematopoietic system where single multipotent cells activate distinct lineage-affiliated gene expression programs prior to exclusive lineage commitment^70^.

Recently, computational systems biology approaches conducted on the transition from naïve to formative pluripotency rather proposed that pluripotency progression does not rely solely upon instructions delivered by few master TFs. It rather appears that a cloud of activity involving multiple coordinated and partially redundant inputs operates to destabilise the existing and self-reinforced naïve gene regulatory network^19^. In that context, the notion of “Molecular Versatility” presented herein might also provide a basis for unifying those principles. We propose that, during pluripotency transition, the stepwise dismantling of the robust naïve TF network, combined with epigenetic remodeling, allows pluripotency TFs to start exerting alternative functions. Therefore, rather than genuine lineage specifiers, pluripotency TFs may be considered as Molecular Versatile actors, and this intrinsic property leads them to exert both pro-self-renewal and pro-lineage commitment functions in closely-related cellular configurations. We detailed this notion below for Oct4 and Nanog.

**Fig. 3:**
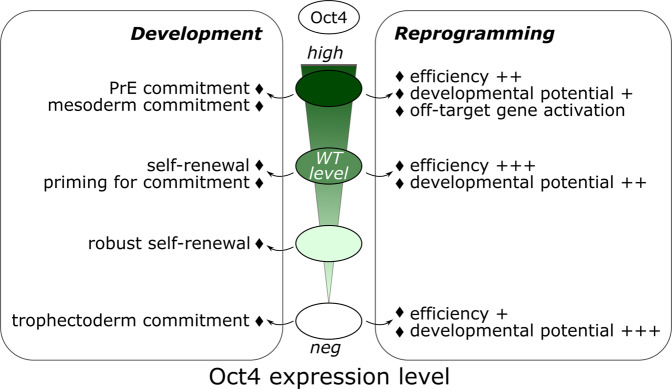
Molecular Versatility of Oct4 during development and reprogramming. The schematic diagram depicts the different functions exerted by the TF Oct4 depending on its expression level. The expression of Oct4 is tightly regulated because gain and loss-of expression respectively triggering differentiation into primitive endoderm/mesoderm or trophectoderm derivatives in ESCs. Unexpectedly, its endogenous level sustains self-renewal but also primes embryonic stem cells for differentiation while a reduced Oct4 level ensures a robust self-renewal state. A precise Oct4 level is also crucial during reprogramming. Its overexpression triggers aberrant gene inductions while iPS cells generated in absence of Oct4 present an unexpected enhanced developmental potential. PrE: Primitive endoderm, WT level: Wild-type level.

The TF *Oct4* (octamer-binding transcription factor 4), also known as POU5F1, promotes (i) self-renewal but also (ii) exit from pluripotency and (iii) endoderm/mesoderm formation during early development^66,71–73^. The comparative analysis of Oct4 genome-wide binding revealed its redistribution on distinct enhancers with and by specific cofactors in ESCs (Klf4, Nanog, Ctcf) and EpiLCs (Otx2, Zic3) but also during endodermal specification (Sox17), indicating how cofactors modulate Oct4 binding and function^74–77^. In addition, Oct4 levels need to be tightly controlled, at both transcriptional and post-transcriptional levels, because slight differences can profoundly alter its function^78^. Reduced Oct4 expression in ESCs was unexpectedly shown to trigger the establishment of a robust self-renewing state in which the Oct4 protein is redistributed on the genome and increasingly bound to enhancer regulatory regions with Nanog^66,71^. However, in a primed context in EpiSCs, reduced Oct4 levels destabilises the pluripotent state and promotes the reversion to a naïve state, indicating differential functions for the same Oct4 dosage^66^. In contrast, at physiological levels, Oct4 triggers the generation of a Nanog-low subpopulation of ESCs that is highly responsive to differentiation cues^66^, in agreement with findings of exogenous Oct4 expression triggering differentiation^79^. The Crispr/Cas9 technology, combined with the recent advances of single-cell approaches, will offer a unique opportunity to revisit these concepts by generating knock-in reporter ESCs for multiple pluripotency TFs and analysing the behaviour of single cells harbouring differential endogenous levels of them. In that sense, a recent report indicated that endogenous elevated OCT4 levels strongly biased ESCs towards both neuroectodermal and mesendodermal fates by increasing chromatin accessibility at differentiation-associated enhancers^80^. Of note, recent evidence also demonstrated the versatility of Oct4 during reprogramming to pluripotency. Silva and colleagues showed first that distinct molecular trajectories to pluripotency required to reach a very precise Oct4 level to be successful^81^. In line with this view, Schöler’s lab refuted the long-standing dogma of a uniquely essential role of Oct4 during reprogramming. Oct4 overexpression was indeed shown to divert cells from a direct route to pluripotency by triggering aberrant gene activation and also, more importantly, to reduce significantly the developmental potential of iPS cells (Fig. 3)^82^.

The TF *Nanog* also harbours strikingly different functions during peri-implantation development. It is well-characterised for its role in the formation of the naïve pluripotent epiblast and the repression of the PrE fate^83,84^. However, it also represses anterior epiblast identity during mouse gastrulation^85^. In addition, Nanog is re-expressed in PGCs and required for germline development in vivo^86^. An elegant study from Surani and colleagues dissected the Molecular Versatility of Nanog. While its exogenous expression triggers naïve ESC self-renewal and resistance to differentiation, it was shown to trigger PGC specification when conducted in primed EpiLCs. Molecularly, the epigenetic resetting occurring in EpiLCs combined with changes in cofactor composition (reduced Sox2 expression), were proposed to allow Nanog to bind and activate enhancers of different sets of genes including the germ cell genes Prdm1 and Prdm14^87^.

Of note, alternative splicing (AS) constitutes another mechanism that allows to redirect TFs during pluripotency progression. A large program of ESC specific-AS events, controlled by the RNA-binding proteins MBNL1 and MBNL2, was described to orchestrate pluripotency progression^88^. Blencowe and colleagues identified in particular a conserved AS event in the Foxp1 transcripts, specifically activated in pluripotent cells, that modifies critical amino acid residues within the forkhead domain and alters its DNA-binding specificity. The two resulting FoxP1 isoforms exert opposite effects by activating pluripotency or differentiation genes, respectively^88–90^.

Altogether, these data indicate a battery of mechanisms by which TFs exert different functions during pluripotency progression.

## Repurpose epigenetic determinants

Epigenetic determinants play a fundamental role during early development and pluripotency progression, as reviewed elsewhere^91^. Recent multiomics profiling of the transition from naïve ESCs to EpiLCs revealed for example major waves of protein phosphorylations but also epigenetic modifications^92^. Moreover, the 3D organization of the chromatin is largely modified during the naïve to primed transition, with the appearance of long-range intra- and inter-chromosomal interactions between H3K27me3 marked genes^93^. Here we present more specifically how epigenetic regulators are repurposed during pluripotency progression. We focus in particular on enhancer and promoter rewiring as well as Trithorax and Polycomb complexes.

### Enhancer reconfigurations

One of the crucial effectors of cell fate transitions is the sequence-dependent binding of TFs to enhancers to regulate gene expression in a cell type-specific manner^94,95^. This is particularly true in the context of pluripotency progression, where the differences in transcriptional output between naïve ESCs and primed EpiSCs are less dramatic than the alterations observed in chromatin profiles at enhancers^74,96,97^. In line with this, different classes of enhancers have been defined in ESCs and EpiSCs, based on varying levels of H3K4me1, H3K27ac, H3K36me3 and pSer2/5 forms of RNA polymerase II^98^. Therefore, a critical challenge for embryonic cells is to ensure a coordinated rewiring of enhancers. We detailed below how Molecular Versatility applies to this process.

**Fig. 4:**
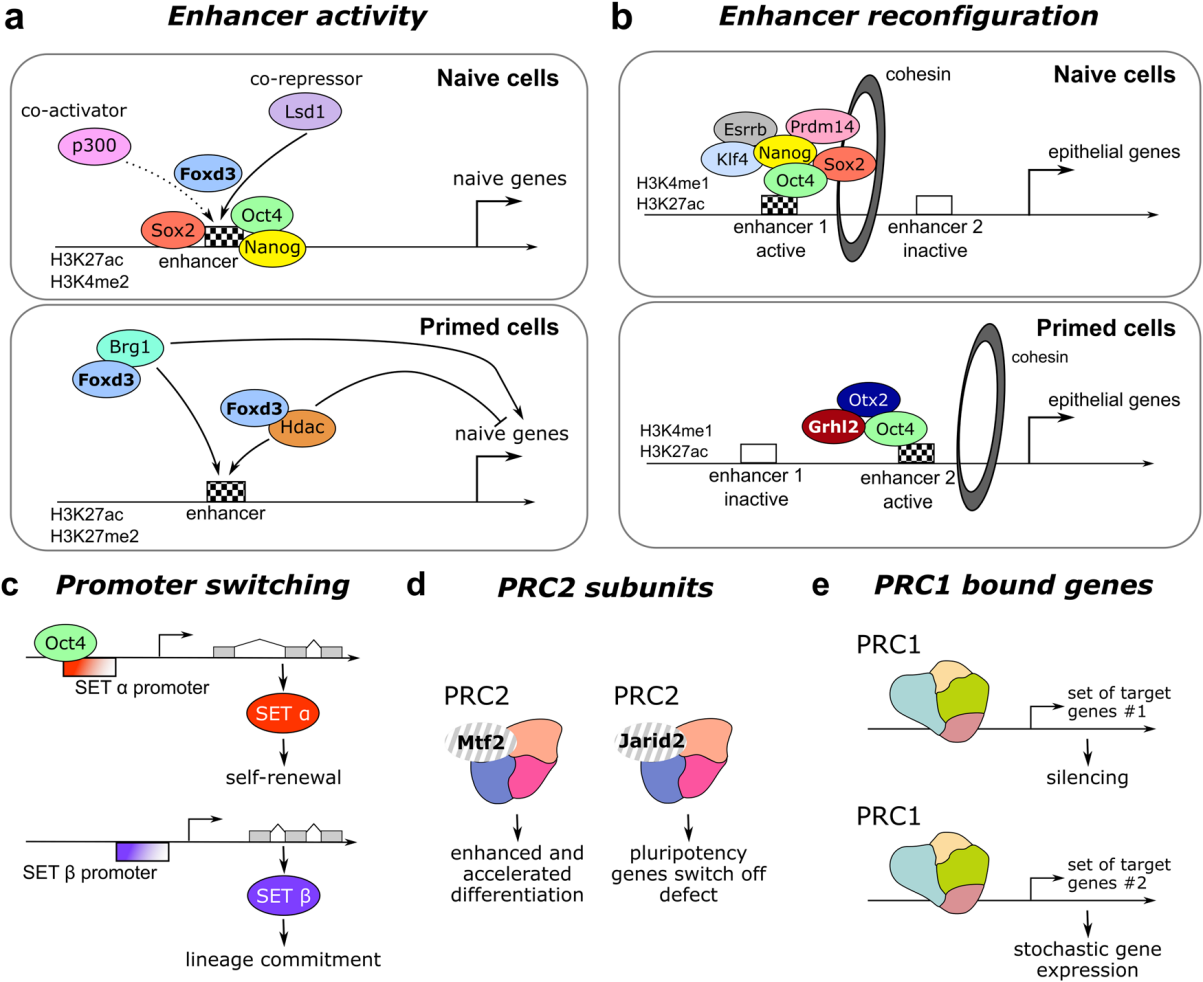
Molecular Versatility of epigenetic mechanisms. **a** The activity of enhancers is controlled by the versatile actions of the FoxD3 transcription factor. In naïve cells, FoxD3 act as a repressor that decommissions active enhancers of naïve pluripotency genes. In primed cells, FoxD3 exerts dual functions by initiating enhancer activity by recruiting Brg1 while simultaneously repressing their maximal activation by recruiting histone deacetylases, illustrating its molecular versatility. **b** The TF Grhl2 orchestrates an enhancer switching program during pluripotency progression. Grhl2 partitions the pluripotency network and sustain the expression of a subnetwork of epithelial genes during naïve-primed transition. **c** Promoter switching for the SET gene in ESCs. This switch leads to the generation of two protein isoforms, SETα and SETβ, that play different functions in ESCs. **d** The subunits of PRC2 dictate different functions for the complex during exit from naïve pluripotency. MTF2 and JARID2 depletion leads to different outcomes during exit from naïve pluripotency. **e** PRC1 has versatile functions depending on the class of genes it binds to. PRC1-bound active genes showed greater cell-to-cell variation when compared with globally active genes.

As a first example, the FoxD3 TF has emerged as a versatile regulator that orchestrates enhancer reconfigurations during exit from naïve pluripotency and progression to the EpiLC state^99^. Foxd3 was initially reported as a repressor that decommissions active enhancers of naïve pluripotency genes via the recruitment of Lsd1 and the reduced binding of co-activators like p300 (Fig. 4a). However, in addition to its repressive role on naïve enhancers, FoxD3 was found to exert other dual functions. It was indeed reported to (i) initiate the activity of newly bound EpiLC-specific enhancers while simultaneously (ii) repress their maximal activation. Molecularly, this is achieved by the co-recruitment of the SWI/SNF chromatin remodeling complex ATPase Brg1 to promote nucleosome removal on enhancers but also of histone deacetylases 1/2 to inhibit their optimal activation^97^ (Fig. 4a).

Interestingly, a complex enhancer switching program was also evidenced during pluripotency progression for genes that are expressed at identical levels in ESCs and EpiSCs, such as Oct4^10,100^. This type of genes was shown to harbour at least 2 classes of enhancers driving their expression in naïve and primed cellular configurations, respectively. Molecularly, this enhancer switching program is controlled by the redistribution of core (Oct4 and Sox2) and stage-specific (Klf4, Prdm14, and Otx2) pluripotency TFs^101^. In more detail, an elegant study by Blelloch and colleagues reported how the TF Grhl2 orchestrates such enhancer switching to partition the pluripotency network and sustain the expression of a subnetwork of epithelial genes during pluripotency progression, notably by regulating Cohesin localisation^101^. As depicted in Fig. 4b, the expression level of the corresponding genes are similar in naïve and primed stem cells but the robustness of the activation is reduced in primed cells to ensure a rapid and efficient silencing later on.

Even if still restricted, these examples highlight how enhancers are rewired during pluripotency progression.

### Promoter reconfigurations

Promoter switching constitutes an additional way to generate Molecular Versatility during pluripotency progression. The use of two alternative promoters led for example to the production of two isoforms of the protein SET (SETα and SETβ) that were found to exert differential effects on stem cell self-renewal and commitment. SETα has beneficial effects on proliferation and linker chaperone activities without interfering with differentiation, while SETβ is essential for proper differentiation (Fig. 4c)^102^. A complex switch between the distal (pLiz) and proximal (pZdbf2) promoters of the Zdbf2 gene has also been described during the naïve-primed transition^103^. The Zdbf2 gene is expressed transiently in the early embryo (and in naïve ESCs) via its pLiz promoter and then from pZdbf2 in somatic cells. Even if the precise function of the protein remains largely unknown, this transient transcription was found to set up an epigenetic state that is required later on and programs postnatal growth. Indeed, mouse embryos deficient for the distal pLiz promoter develop normally but completely fail to activate Zdbf2 in the postnatal brain and show reduced growth. Therefore, even if this promoter switch appears dispensable for embryogenesis, it signals essential regulatory information in the adult^104^. At the molecular level, the transient transcription from pLiz promotes de novo DNA methylation upstream of the pZdbf2 promoter, which antagonises Polycomb-mediated repression. Interestingly, the two promoters use similar enhancers but their respective activity in ESCs and EpiLCs is controlled by contacts mediated by the CTCF protein. The alternative usage of enhancers/promoters represents an elegant example of Molecular Versatility that contributes to sustain stemness but prepares the genome for a robust and coordinated progression towards gastrulation.

### Trithorax (TrxG) complexes

TrxG complexes mediate the deposition of the active histone mark H3K4 methylation via their catalytic subunits, the histone methyltransferases SET/MLL^105^. TrxG complexes display versatile functions in ESCs, namely coordinating (i) self-renewal or (ii) lineage commitment, depending on the composition of subunits. The core member subunit Wdr5 was described to promote naïve ESCs self-renewal by maintaining global H3K4me3 levels and by cooperating with the Oct4/Nanog/Sox2 circuitry, sharing target genes and regulatory functions^106^. Ash2l, another core subunit, is also required for pluripotency: Ash2l knockdown in ESCs leads to differentiation and derepression of mesodermal markers^107^. Conversely, the depletion of the subunit Dpy-30 does not impact ESCs self-renewal but rather impairs their differentiation, particularly along the neural lineage. This phenotype is largely mediated by the erasure of the H3K4me3 mark at bivalent promoters that induces the dissolution of their poised state^108^. Interestingly, the MLL1 subunit was on the contrary reported to maintain the identity of primed EpiSCs. MLL1 inhibition leads to a global redistribution of the H3K4me1 mark and therefore to a profound rewiring of the enhancer landscape, leading to the spontaneous reversion of primed EpiSCs into naïve ESCs^109^.

### Polycomb repressive complexes

The PRC complexes (PRC2 and PRC1), formed by Polycomb group proteins, play essential roles in pluripotency and development by mediating chromatin modifications. PRC2 is recruited to specific genomic locations and catalyzes deposition of H3K27me3, which in turn recruits PRC1, resulting in the generation of H2AK119ub1 and transcriptional repression. In ESCs, PRC1/2 co-occupy indeed regions marked with H3K27me3, with a large proportion of these sites being proximal to genes of lineage commitment^110,111^. Accordingly, PRC2- (Eed KO) and PRC1- (Ring1A/B KO) deficient ESCs showed increased expression of differentiation genes^110,112^. However, PRC function is far more broad and versatile during pluripotency progression than initially established^113^.

First, recent integrative proteomic profiling of ground state ESCs revealed a critical function for PRC2 in globally shaping the pluripotent epigenome to avoid the acquisition of primed features such as DNA methylation^114^. In ground state ESCs, PRC2 and H3K27me3 are indeed widely distributed on both euchromatin and heterochromatin, when compared with Serum/LIF ESCs.

Second, a recent body of work demonstrated the versatile functions exerted by the different subunits of PRC2. By combining single-cell transcriptomics and embryoid body formation, Loh et al. showed that the depletion of the two distinct PRC2 subunits MTF2 and JARID2 led to different phenotypes during exit to naïve pluripotency. MTF2 absence enhanced and accelerated differentiation towards the three lineages, as expected, but JARID2 null ESCs struggled to turn off pluripotency genes and were predominantly converted into early differentiating precursors, with reduced efficiency towards the mesendoderm lineages (Fig. 4d)^115^.

Moreover, another study highlighted the critical role of PRC2 in promoting the specification of the ectoderm in rodent and human primed stem cells^116^. Targeting PRC2 genes in human ESCs led to pluripotency loss and to spontaneous differentiation towards a meso-endoderm fate. The attempts to convert mouse PRC2-deficient naïve ESCs into primed EpiSCs failed and led to similar differentiation defects as human cells. Even if the precise molecular mechanisms remain to be elucidated, these versatile functions of PRC2 might be attributable to (i) the coordinated relocation of various PRC2 complexes and to (ii) different thresholds for activation of various classes of genes.

PRC1 was also reported to promote both (i) self-renewal and (ii) lineage commitment in ESCs, with reports highlighting the importance of the composition of its subunits in these versatile functions^117,118^. In naïve ESC, Cbx7, which is the main PRC1-associated expressed Cbx protein, is required to maintain the undifferentiated state in a robust manner. This Cbx7-containing PRC1 complex represses the transcription of differentiation genes and of the other subunits Cbx2 and Cbx4. Once induced during differentiation, Cbx2- and Cbx4-containing complexes are redistributed to repress a large set of genes, including pluripotency-related loci. In addition, Cbx2/4 deficiency was demonstrated to skew in vivo teratoma differentiation towards the endodermal fate, indicating a specific function in germ layer formation, as also revealed for the other PRC1 subunits Ring1B and Pcgf6^119,120^. Thus, Cbx proteins confer distinct target selectivity and functions to the canonical PRC1 complex. In addition, variant PRC1 complexes, that are recruited to target genes mainly independently of PRC2 and H3K27me3, were found recently to be mainly responsible for gene repression in ESCs^121,122^.

Moreover, although PRCs are known to exert a repressive effect, interestingly, the cohort of PRC-bound genes contains not only silent genes, but also genes with intermediate and high expression^123^. Recent single-cell RNA-seq analyses showed that active genes bound by PRC in ESCs are subjected to a switch between active and repressed states and harbour therefore greater cell-to-cell variation than classical active genes. This phenomenon, by favoring stochastic expression of genes, may play a key role in stem cell physiology (Fig. 4e)^124,125^. In the same line, PRC1 complexes containing Cbx8 are recruited transiently on differentiation genes to facilitate their activation^126^. Similarly, the PRC1 subunit Mel18, crucial for the repression of differentiation genes in ESCs, facilitates the expression of TFs essential for cardiac differentiation in progenitors^127^. Altogether, these findings support the versatile functions and modes of action of PRC during early development.

## A tight control of molecular versatility

Altogether, the previous sections highlighted how molecules are accurately redeployed to exert different, sometimes opposite, functions in closely-related stem cell configurations. However, in order for the changes to occur at the correct time and place, Molecular Versatility must be tightly regulated and connected with the microenvironment to ensure channelling and robustness of pluripotency progression^4^. Signalling pathways are known to be directly connected to pluripotency TFs. Effectors such as Stat3 and Smad2/3 co-occupy the ESC genome with the TFs Oct4, Nanog and Sox2, providing a direct rationale for the connection between extrinsic cues, signalling and the TF network^128–130^. However, certain signalling pathways were recently found to directly control the Molecular Versatility of TFs. For instance, the TF Etv5 was reported to undergo a switch in its function depending on ERK1/2 signalling activity. Indeed, Etv5 supports ESC self-renewal when ERK1/2 is inhibited but promotes exit from naïve pluripotency when ERK1/2 is active^17^. As another example, the EMT-TF Snail1 was found to control metabolic cascades in ESCs in a WNT-independent manner. However, during ESC commitment, Snail1 is induced by WNT to regulate exit from primed pluripotency and neuroectodermal fate emergence^131^.

In order to be precisely regulated, the activity of versatile factors was also found to be controlled by multiple signals. As a first example, we reported that the Netrin-1 signalling pathway regulates naïve pluripotency by modulating the responsiveness of embryonic cells to both FGF and WNT signals^25,132^. This dual function is achieved via the induction of complex signalling cascades downstream of the two Netrin-1 receptors Unc5b and Neo1. However, the expression of the *Netrin-1* transcript itself is also directly and antagonistically controlled by the WNT and FGF/MAPK pathways, positioning this molecule as an integrated molecular sensor for the embryonic cell.

The activity of basic helix-loop-helix (bHLH) TFs is also controlled by multiple signals during pluripotency progression. For instance, Tcf15 was described to prime pluripotent cells for differentiation in response to FGF/MAPK signalling^133^. However, while Tcf15 transcription is directly induced by FGF/MAPK, its biological activity is suppressed by Id proteins that are under the control of BMP signalling^22^. Therefore, Tcf15 is able to molecularly sense multiple external cues (FGF/BMP) to finely tune its activity and to confer accuracy to the naïve-primed transition. Consistently, Tcf15 expression is transient in the early embryo, readily detectable at E4.5 but turned off by E5.5. The bHLH TF Tfe3, that promotes naïve pluripotency, is rapidly excluded from the nucleus of ESCs by the mTORC1 and lysosome signalling^134,135^, highlighting an alternative way to precisely integrate signalling cues.

Another elegant study from Lowell et al. deciphered how the BHLH TF Id1 preserves pluripotency and avoids premature differentiation during the naïve-primed transition by being multiply regulated. Id1 expression is induced when embryonic cells lose Nanog expression but have not yet acquired Nodal activity. The Id1 protein suppresses FGF/MAPK signalling in order to protect these Nanog-negative cells from differentiation. Then, once Nodal activity is operating in these transitioning cells, Nodal itself suppresses Id1 expression and leads to a rise in to FGF activity contributing to sustaining pluripotency in primed cells^136^.

## Concluding remarks

**Fig. 5:**
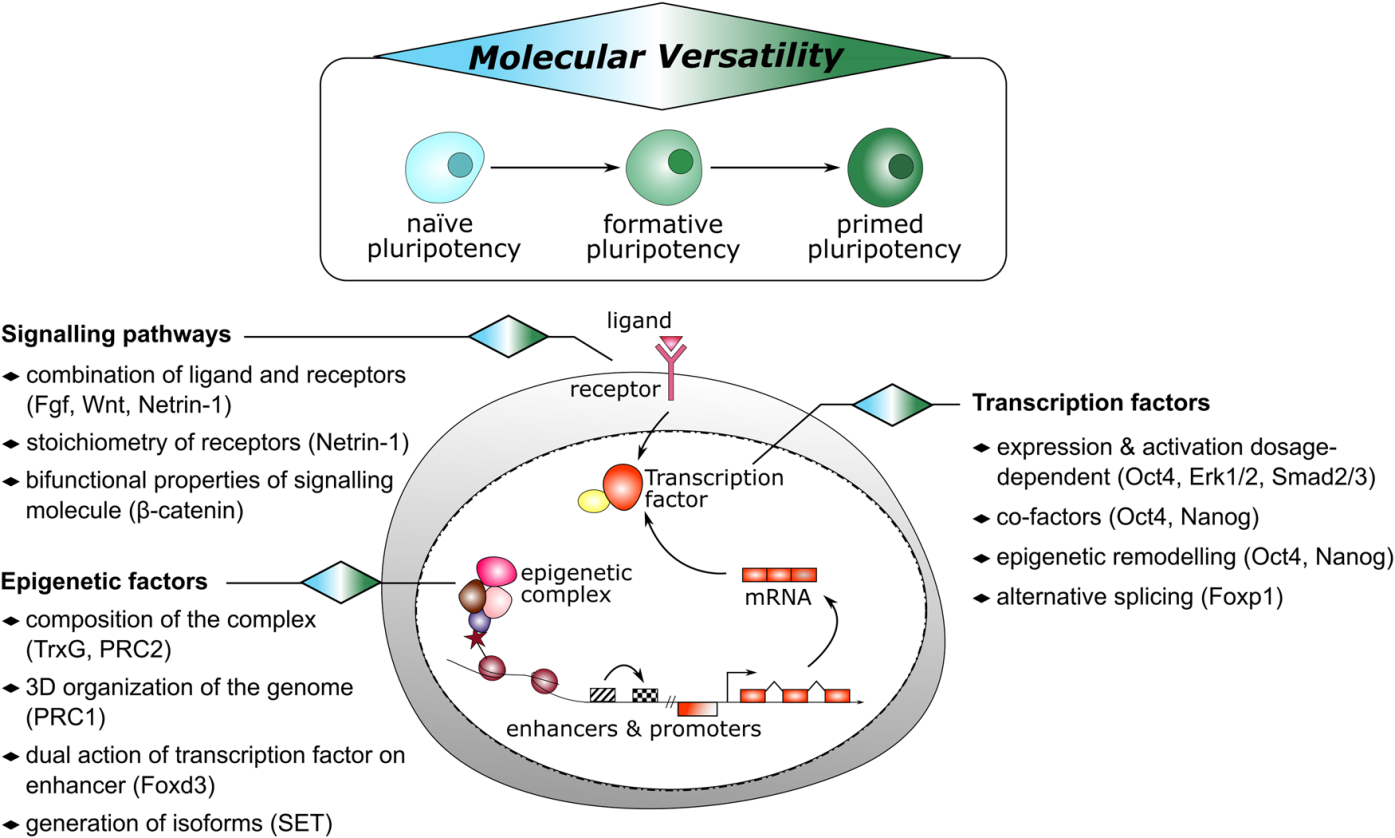
Molecular Versatility during pluripotency progression. The scheme summarises the different mechanisms highlighted in the Perspective to allow molecules (signalling pathway, TF and epigenetic factors) to exert different functions in closely-related stem cell configurations.

This perspective presents converging findings demonstrating that a large repertoire of molecules are repurposed to exert different, sometimes opposite, functions in closely-related stem cell configurations during pluripotency progression. We propose to regroup these multiple molecular mechanisms by which signalling pathways, TFs and epigenetic regulators are rapidly rewired with the notion of Molecular Versatility, as summarised in Fig. 5. We believe that these redeployments, by acting in concert with profound transcriptomic and epigenomic rewirings, contribute to ensure a robust and coordinated programming of embryonic stem cell fate in response to extrinsic signals. In addition, we propose that Molecular Versatility may confer a certain degree of reversibility and flexibility to the developmental transition processes by involving molecules already present within the embryonic cells. Because the examples used in this Perspective are mainly based on the comparison of naïve ESCs and primed EpiLCs/EpiSCs, it remains to be investigated whether and how Molecular Versatility takes place dynamically in truly transitioning cells. The recent capture of intermediate states such as Rosette and Formative stem cells (RSC and FS respectively), combined with the power of computational biology, will certainly help in better defining these relatively underexplored layers of regulation. Moreover, the advances in the development of single cell-based approaches will also permit to address precisely how these mechanisms take place in transitioning cells. In that sense, pluripotency progression provides an amenable experimental system for dissection of stem cell fate transition paradigm, that will surely bring highly valuable concepts for the biology of embryonic but also adult normal and pathological stem cells.
